# LncRNA DCST1-AS1 Promotes Endometrial Cancer Progression by Modulating the MiR-665/HOXB5 and MiR-873-5p/CADM1 Pathways

**DOI:** 10.3389/fonc.2021.714652

**Published:** 2021-08-23

**Authors:** Jie Wang, Pingping Shi, Huaixiang Teng, Lixiang Lu, Hailong Guo, Xiuqin Wang

**Affiliations:** ^1^Gynaecology Clinic, People’s Hospital of Rizhao, Rizhao, China; ^2^No. 2 Department of Gynaecology, People’s Hospital of Rizhao, Rizhao, China; ^3^Reproductive Medicine Center, Maternal and Child Health Hospital of Rizhao, Rizhao, China; ^4^No. 2 Department of Gynaecology, Baiqiuen Hospital of Rizhao, Rizhao, China

**Keywords:** endometrial cancer, long noncoding RNA, DC-STAMP domain-containing 1-antisense 1, microRNA-665, homeobox B5, microRNA-873-5p, cell adhesion molecule 1

## Abstract

Dysregulation of long noncoding RNA (lncRNA) is implicated in the initiation and progression of various tumors, including endometrial cancer (EC). However, the mechanism of lncRNAs in EC tumorigenesis and progression remains largely unexplored. In this work, we identified a novel lncRNA DC-STAMP domain-containing 1-antisense 1 (DCST1-AS1), which is highly upregulated and correlated with poor survival in EC patients. Overexpression of DCST1-AS1 significantly enhanced EC cell proliferation, colony formation, migration, and invasion *in vitro* and promoted tumor growth of EC *in vivo*. Mechanistically, DCST1-AS1 mediated EC progression by inducing the expression of homeobox B5 (HOXB5) and cell adhesion molecule 1 (CADM1), *via* acting as a competing endogenous RNA for microRNA-665 (miR-665) and microRNA-873-5p (miR-873-5p), respectively. In addition, we found that the expression of miR-665 and miR-873-5p was significantly downregulated, while HOXB5 and CADM1 expression levels were increased in EC tissues. Taken together, our findings support the important role of DCST1-AS1 in EC progression, and DCST1-AS1 may be used as a prognostic biomarker as well as a potential therapeutic target for EC.

## Introduction

Endometrial cancer (EC) is the most common malignant gynecological cancer in women (1, 2). Most women diagnosed with EC have the early-stage disease and show favorable outcomes (3, 4). However, there is a subset of ECs in which metastasis and recurrences do occur (3, 4). Clinical outcomes worsen considerably for women diagnosed with clinically aggressive disease (5). Thus, there is an urgent need to develop more effective strategies for the diagnosis and treatment of EC.

Long noncoding RNAs (lncRNAs) represent a large class of nonprotein-coding transcripts larger than 200 nucleotides (6, 7). Numerous lncRNAs are aberrantly expressed in a broad spectrum of cancers, and they play important roles in regulating gene expression (6, 7). LncRNAs could act as guides to promote or inhibit transcription and as scaffolds by interacting with chromatin-modifying complexes (8). Furthermore, lncRNAs function as competing endogenous RNAs (ceRNAs) to sponge microRNAs (miRNAs), indirectly modulating gene expression (7). In human tumors, lncRNAs are considered as regulators of multiple cancer phenotypes, including tumor cell proliferation, motility, invasion, and metastasis (9). For example, lncRNA small nucleolar RNA host gene 5 (SNHG5) sponged miR-25-3p to enhance B‐cell translocation gene 2 (BTG2) expression, thereby repressing EC cell proliferation (10). LncRNA nuclear paraspeckle assembly transcript 1 (NEAT1) was found to promote EC cell growth and invasion through its interaction with miR-144-3p or targeting miR-361 (11, 12). Additionally, lncRNA titin-antisense RNA1 (TTN−AS1) and lncRNA taurine-upregulated gene 1 (TUG1) were demonstrated to promote EC progression by sponging miRNAs (13, 14). Furthermore, another lncRNA steroid receptor RNA activator (SRA) was considered to promote EC progression by activating the Wnt signaling (15).

LncRNA DC-STAMP domain-containing 1-antisense 1 (DCST1-AS1) was overexpressed in various tumor types, including breast cancer, glioblastoma, cervical cancer, and hepatocellular carcinoma (16–19). Elevated expression of DCST1-AS1 regulates cancer progression by sponging different miRNAs and influencing the downstream signaling pathways (16–19). However, our current understanding of DCST1-AS1 in EC is still limited. Recently, DCST1-AS1 was demonstrated to promote EC cell invasion by sponging miR-92a-3p and upregulating the expression of Notch1 (20). The function and mechanism of DCST1-AS1 involved in regulating EC progression remain unexplored.

In this study, we predicted that DCST1-AS1 potentially interacts with microRNA-665 (miR-665) and microRNA-873-5p (miR-873-5p), which play important roles in a series of cancers acting as either oncogene or repressor (21–23). Our study demonstrated that DCST1-AS1 was upregulated in EC tissues and its expression was associated with worse patient outcomes. DCST1-AS1 could promote EC progression by binding with miR-665 and miR-873-5p and inducing the expression of homeobox B5 (HOXB5) and cell adhesion molecule 1 (CADM1), respectively. Therefore, we revealed a novel mechanism by which lncRNA DCST1-AS1 facilitated EC proliferation *via* the miR-665/HOXB5 axis and the miR-873-5p/CADM1 axis. Thus, these signaling pathways might be potential targets for developing therapeutic strategies for the treatment of EC.

## Materials and Methods

### Patient Tissue Specimens

Seventy pairs of human EC tissues and corresponding adjacent normal tissues were obtained from patients treated in People’s Hospital of Rizhao. All patients signed the informed consent form for the use of samples, and this study was approved by the Ethical Committee on Scientific Research of People’s Hospital of Rizhao. All tissue samples were immediately frozen in liquid nitrogen after surgical removal and stored at −80°C.

### Cell Culture and Transfection

Four human EC cell lines (HEC-1A, HEC-1B, RL-95-2, and JEC) and human normal endometrial stromal cells (HESCs) were obtained from the ATCC. Cells were cultured in Dulbecco’s modified Eagle’s medium (DMEM) (Gibco, Carlsbad, CA, USA) supplemented with 10% fetal bovine serum (FBS, Gibco) at 37°C in a 5% CO_2_ incubator. Two small interfering RNAs (siRNAs) targeting lncRNA DCST1-AS1, negative control siRNA (si-NC), miR-665 mimic, miR-873-5p mimic, control mimic (miR-NC), miR-665 inhibitor, miR-873-5p inhibitor, control inhibitor, pcDNA-HOXB5, pcDNA-CADM1, and empty vector (pcDNA3.1) were synthesized by GenePharma Co., Ltd. (Shanghai, China). Briefly, EC cells were seeded into six-well plates at 70% cell confluence. Cell transfection was conducted using Lipofectamine 2000 (Invitrogen, Carlsbad, CA, USA) according to the protocol of the manufacturer. After transfection, EC cells were cultured for the indicated time and subjected to the following experiments.

### RNA Extraction and Quantitative Real-Time PCR

Total RNA was extracted from frozen tissues or cell lines using TRIzol reagent (Invitrogen), and then was reverse transcribed into complementary (cDNA) using PrimerScript RT reagent (TaKaRa, Dalian, China). The quantitative real-time PCR (qRT-PCR) assay was conducted using SYBR Green Mix (TaKaRa) on a Bio-Rad system. GAPDH was used as the internal control. The primer sequences for qRT-PCR are listed in **Table 1**.

**Table 1:** The primers sequence for qRT-PCR assay.

Gene	Forward	Reverse
*LncRNA DCST1-AS1*	TTCGTCTGGTCCCAATGTGTGG	AAGCAGGACGAGTAAACCAACC
*U6*	GCTTCGGCAGCACATATACTAAAAT	CGCTTCACGAATTTGCGTGTCAT
*miR-665*	GCCGAGACCAGGAGGCUGA	CTCAACTGGTGTCGTGGA
*miR-873-5p*	GCAGGAACUUGUGAGUCUCCU	AGGAGACUCACAAGUUCCUGC
*GADPH*	GGAGCGAGATCCCTCCAAAAT	GGCTGTTGTCATACTTCTCATGG
*HOXB5*	TGCATCGCTATAATTCATT	GCCTCGTCTATTTCGGTGA
*CADM1*	TCAACACGCCGTACTGTCTG	GTGGGAGGAGGGATAGTTGTG

### Cell Counting Kit-8 Assay

Cell viability experiment was conducted using a Cell Counting Kit-8 (CCK-8) assay (Dojindo Laboratories, Kumamoto, Japan). Briefly, cells were transfected and seeded into 96-well plates. Twenty-four, 48, and 72 h later, the CCK-8 solution was added to each well and incubated for 4 h at 37°C. The absorbance of each well was measured at 450 nm wavelength using a microplate reader (PerkinElmer, Shanghai, China). All assays were performed in triplicate.

### Colony Formation Assay

EC cells were seeded on six-well plates and maintained in the culture media at 37°C for 2 weeks. Then, cells were fixed with 4% paraformaldehyde for 10 min and stained using 0.1% crystal violet solution for 30 min at room temperature. The number of colonies was counted using ImageJ software.

### Cell Migration and Invasion Assay

The transfected cells were seeded in the top chamber in 200 µl of serum-free DMEM medium (Gibco) with Matrigel (BD Biosciences, San Jose, CA, USA), and a complete medium (750 µL) containing 10% FBS was added to the lower chamber. After 24 or 48 h, the migrated or invaded cells were fixed and stained with crystal for 15 min at room temperature. Five random fields per well were observed, and the cells were counted under the microscope.

### Flow Cytometry Analysis

The apoptosis of EC cells was examined using an Annexin V-FITC/propidium iodide (PI) staining assay (BD Biosciences). After washing with cold PBS, the cells were resuspended in binding buffer followed by staining with Annexin V-FITC/PI at room temperature for 15 min in the dark. Apoptotic cells were evaluated by a fluorescence-activated cell sorting (FACS) flow cytometer (BD Biosciences). All experiments were performed in triplicate.

### Luciferase Reporter Assay

The wild-type (WT) fragments of DCST1-AS1 3′-untranslated region (UTR), HOXB5 3′-UTR, and CADM1 3′-UTR were synthesized and cloned into pGL3 vector (Promega Corporation, Madison, WI, USA).

Mutations (MUT) of the miRNA-binding sites in the DCST1-AS1, HOXB5 3′-UTR, and CADM1 3′-UTR were generated using a QuickChange site-directed mutagenesis kit (Stratagene, La Jolla, CA, USA). Then, miR-665 mimic, miR-873-5p mimic, or control mimic was co-transfected with the indicated reporter plasmids into EC cells using Lipofectamine 2000 (Invitrogen). Luciferase assays were performed using the Dual-Luciferase Reporter Assay System (Promega) according to the instructions of the manufacturer.

### Tumor Xenograft Studies

Animal experiments were approved by the Institutional Animal Care and Use Committee of the People’s Hospital of Rizhao. Four- to 6-week-old female nude mice were purchased from the Shanghai Laboratory Animal Center of the Chinese Academy of Sciences (Shanghai, China) and were randomly divided into two groups for further study. We established stable EC lines using shRNA against lncDCST1-AS1 or control shRNA. EC cells with stable knockdown of lncDCST1-AS1 or control cells were injected subcutaneously into the flanks of nude mice. Tumor volume was calculated as length (mm) × width^2^ (mm^2^) × 0.5. After 40 days, tumors were harvested and weighed.

### Bioinformatics

The expression level of lncRNA DCST1-AS1, miR-665, miR-873-5p, HOXB5, and CADM1 was examined using the Encyclopedia of RNA Interactomes (ENCORI) database (http://starbase.sysu.edu.cn/). The online website tool DIANA-TarBase (https://carolina.imis.athena-innovation.gr/diana_tools/web/index.php) was used to predict the interaction between microRNAs, DCST1-AS1, and mRNAs.

### Statistical Analysis

All data were presented as means ± standard deviation. Student’s *t*-test was used to compare the difference between the two groups. One-way analysis of variance (one-way ANOVA) was used to analyze the differences among multigroups. All of the statistical calculations were performed using GraphPad Prism 5 software (San Diego, CA, USA). *P <*0.05 was considered statistically significant: *P < 0.05, **P < 0.01, and ***P < 0.001.

## Results

### LncRNA DCST1-AS1 Is Overexpressed in EC Tissues and Cell Lines

**Figure 1:**
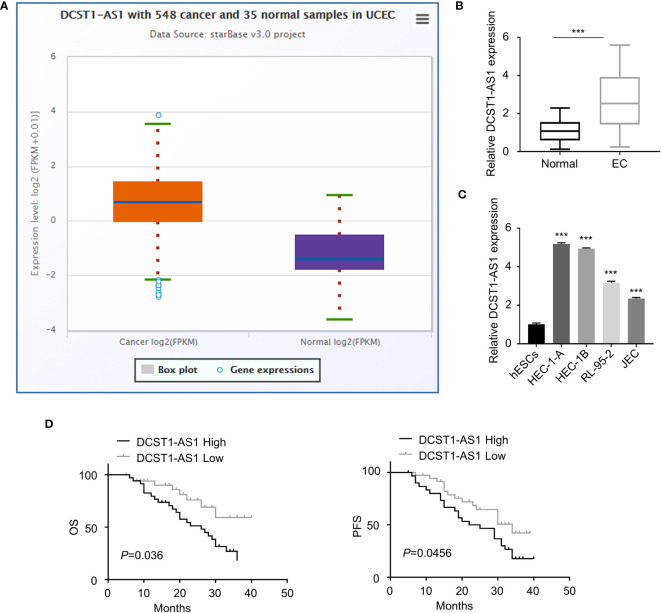
LncRNA DC-STAMP domain-containing 1-antisense 1 (DCST1-AS1) expression is overexpressed in endometrial cancer (EC) tissues and cell lines. **(A)** The expression of lncRNA DCST1-AS1 in 548 EC tissues and 35 normal endometrium tissues (ENCORI database). **(B)** qRT-PCR analysis of DCST1-AS1 expression in 70 EC tissues and matched normal tissues. **(C)** Comparison of lncRNA DCST1-AS1 expression in the indicated EC cell lines and human normal endometrial stromal HESC cells using the qRT-PCR assay. **(D)** Kaplan–Meier curves for overall survival (OS) (left) and progression-free survival (PFS) (right) in EC patients with low or high DCST1-AS1 expression. ***P < 0.001.

We first compared the expression of lncRNA DCST1-AS1 in normal endometrial tissues (*n* = 35) and EC tissues (*n* = 548) using the online database ENCORI. As shown in **Figure 1A**, lncRNA DCST1-AS1 was significantly upregulated in EC tissues compared with normal endometrium tissues. Our qRT-PCR analysis of lncRNA DCST1-AS1 expression from 70 pairs of EC tissues and adjacent normal tissues showed that lncRNA DCST1-AS1 was overexpressed in EC patients (**Figure 1B**). Consistently, the expression of lncRNA DCST1-AS1 was significantly higher in EC cell lines than that in human normal endometrial stromal HESC cells (**Figure 1C**). Furthermore, 70 EC patients were classified into lncRNA DCST1-AS1 low and lncRNA DCST1-AS1 high groups. The results revealed that the expression of DCST1-AS1 was not related to the age and gender of the patients, but higher expression of DCST1-AS1 was significantly associated with larger tumor size, advanced TNM stage, and lymph node metastasis (**Table 2**).

**Table 2:** Correlation between lncRNA DCST1-AS1 expression and clinicopathological factors in EC.

Factor		DCST1-AS1 expression		*P*-value
		Low (*n* = 35)	High (*n* = 35)	
Age				0.3388
	≤50	15	19	
	>50	20	16	
Sex				1
	Male	0	0	
	Female	35	35	
Tumor size				0.0168
	≤3 cm	23	13	
	>3 cm	12	22	
T classification				0.026
	T1–T2	21	17	
	T3–T4	14	18	
N classification				0.008
	N0–N1	22	11	
	N2–N3	13	24	
Lymph node metastasis				0.0303
	No	24	15	
	Yes	11	20	

Survival analysis using the Kaplan–Meier (KM)-plotter database suggested that those patients with high DCST1-AS1 expression had worse overall survival (OS) and progression-free survival (PFS) than those with low DCST1-AS1expression (**Figure 1D**). All these results suggested that DCST1-AS1 is overexpressed in EC and correlated with poor clinical outcomes.

### Depletion of LncRNA DCST1-AS1 Inhibits EC Cell Proliferation, Migration, and Invasion

**Figure 2:**
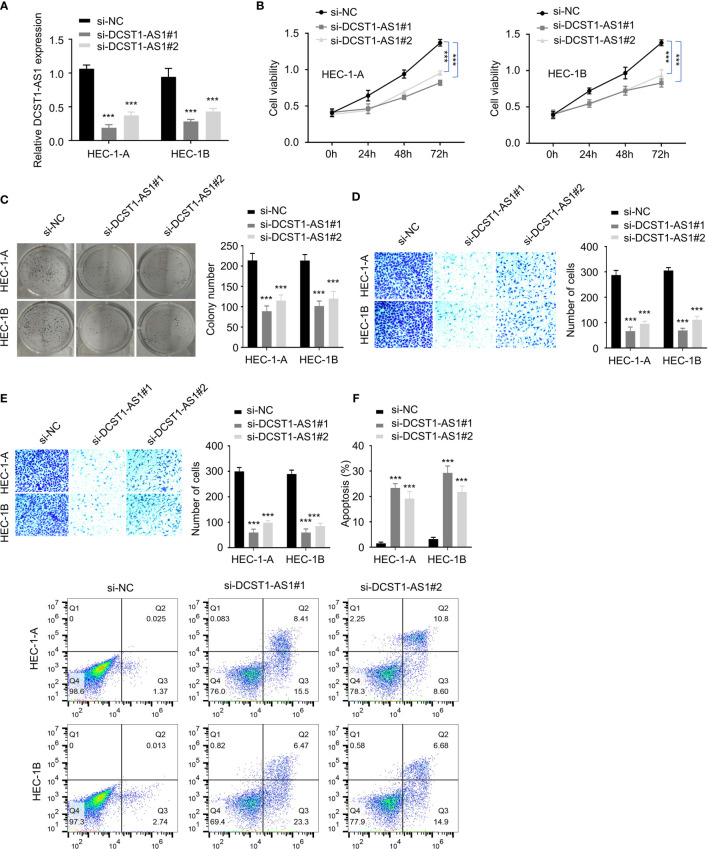
Depletion of lncRNA DCST1-AS1 inhibits EC cell proliferation, migration, and invasion. **(A)** The expression of lncRNA DCST1-AS1 was measured by RT-qPCR assay in HEC-1A and HEC-1B cells transfected with DCST1-AS1 siRNA (si-DCST1-AS1) or control siRNA (si-NC). **(B)** Cell Counting Kit-8 (CCK-8) assay showed that depletion of DCST1-AS1 suppressed EC cell proliferation. **(C)** Colony formation assay of EC cells transfected with si-NC or si-DCST1-AS1. **(D, E)** Transwell migration and invasion assay showed that DCST1-AS1 knockdown inhibited EC cell migration **(D)** and invasion **(E)**. **(F)** Flow cytometry assay showed that DCST1-AS1 knockdown increased EC cell apoptosis. ***P < 0.001.

To investigate the role of lncRNA DCST1-AS1 in EC, we silenced the expression of lncRNA DCST1-AS1 in EC cell lines using siRNAs and examined the effect of DCST1-AS1 knockdown on EC cell proliferation, migration, and invasion. First, we confirmed the transfection efficiency by qRT-PCR analysis (**Figure 2A**). Then, CCK-8, colony formation, migration, and invasion assays suggested significant inhibition of proliferation, growth, migration, and invasion of EC cells transfected with DCST1-AS1 siRNA compared with those transfected with control siRNA (**Figures 2B–E**). Moreover, silencing of lncRNA DCST1-AS1 enhanced cell apoptosis (**Figure 2F**). Together, these results suggested that lncRNA DCST1-AS1 functions as an oncogenic lncRNA in EC.

### LncRNA DCST1-AS1 Acts as a Sponge for miR-665 and miR-873-5p

**Figure 3:**
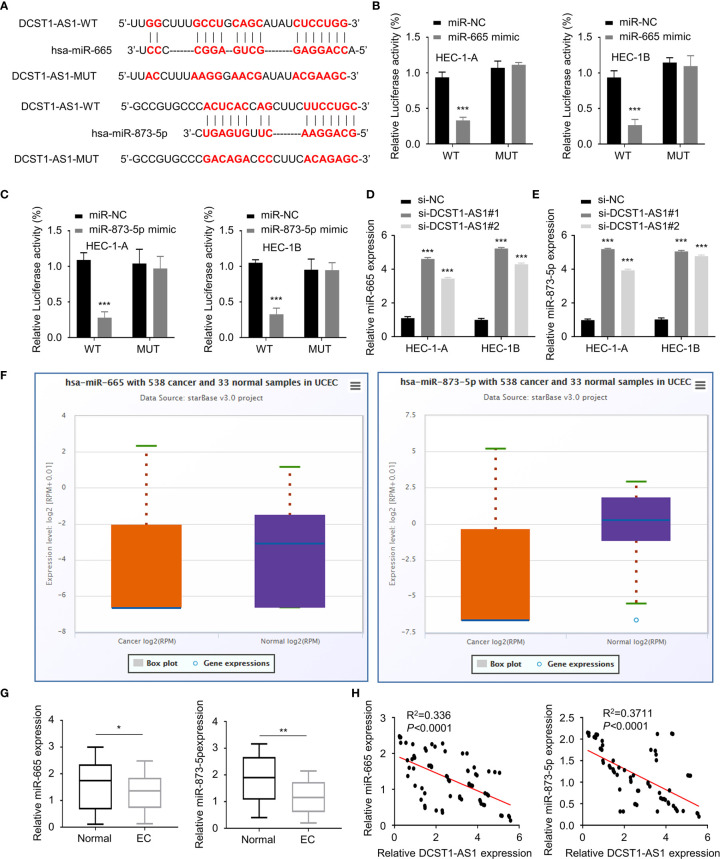
LncRNA DCST1-AS1 acts as a sponge for microRNA-665 (miR-665) and microRNA-873-5p (miR-873-5p). **(A)** Predicted and mutant (lower panel) sequence alignments of miR-665 and miR-873-5p with DCST1-AS1. **(B)** Wild-type (WT) DCST1-AS1 and mutant (MUT) DCST1-AS1 luciferase reporters were co-transfected with miR-665 mimic or control mimic (miR-NC). Luciferase activity was detected using luciferase assays. **(C)** Luciferase reporter assays showed that miR-873-5p significantly reduced luciferase activity of WT DCST1-AS1, but mutation of the miR-873-5p binding site abrogated the inhibitory effects of miR-873-5p. **(D, E)** The levels of miR-665 **(D)** and miR-873-5p **(E)** were tested by RT-qPCR in EC cells with (or without) DCST1-AS1 depletion. **(F)** The expression of miR-665 and miR-873-5p in EC (*n* = 538) and tumor-adjacent normal tissues (*n* = 33) (ENCORI database). **(G)** The levels of miR-665 and miR-873-5p in EC tissues and the adjacent normal tissues were measured using qRT-PCR assay. **(H)** The correlation of DCST1-AS1 and miR-665/miR-873-5p expression was analyzed by Spearman’s correlation analysis. *P < 0.05, **P < 0.01, ***P < 0.001.

To explore how DCST1-AS1 exerts its function, we predicted miRNAs that can bind with DCST1-AS1 using the online database. The results showed that DCST1-AS1 contains the putative binding sites for miR-665 and miR-873-5p (**Figure 3A**). Using luciferase reporter assays, we showed that overexpression of miR-665 and miR-873-5p reduced the luciferase activity of the wild-type DCST1-AS1 reporter gene, but not the mutant DCST1-AS1 reporter gene (**Figures 3B, C****)**.

Furthermore, we found that silencing of lncRNA DCST1-AS1 increased the expression of miR-665 and miR-873-5p (**Figures 3D, E****)**. We tested the expression of miR-665 and miR-873-5p in EC tissues and normal tissues using the ENCORI database. We demonstrated that the expression of both miR-665 and miR-873-5p was significantly downregulated in EC samples (**Figures 3F, G****)**. The qRT-PCR assays showed that the expression of DCST1-AS1 was negatively correlated with miR-665 and miR-873-5p expression (**Figure 3H**). These results indicated that DCST1-AS1 directly sponges miR-665 and miR-873-5p in EC cells.

### LncRNA DCST1-AS1 Sponges miR-665 to Upregulate HOXB5 Expression

**Figure 4:**
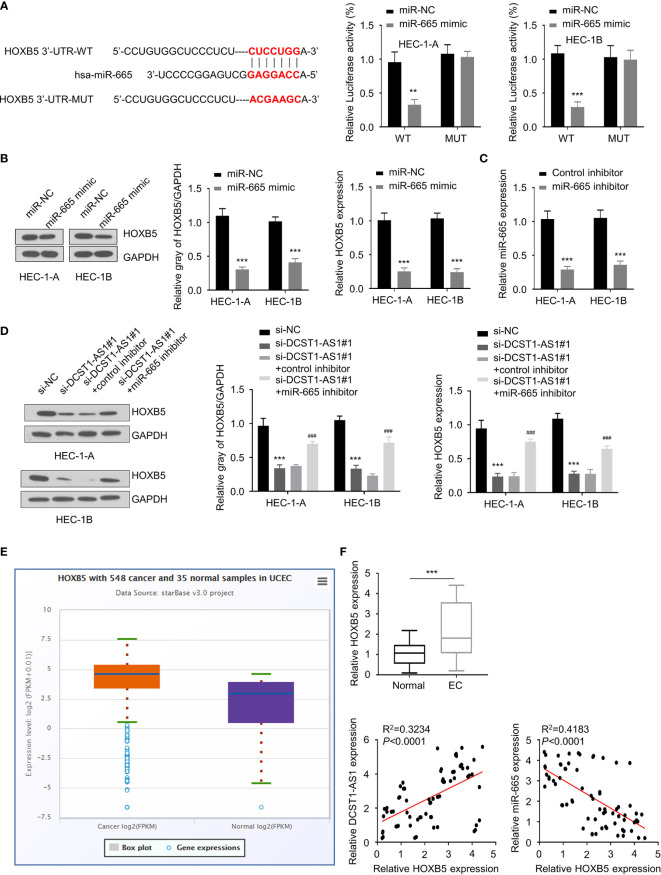
LncRNA DCST1-AS1 sponges miR-665 to upregulate homeobox B5 (HOXB5) expression. **(A)** Predicted and mutant (lower panel) sequence alignments of miR-665 with HOXB5 3′-UTR sequence. Luciferase reporter assays showed that miR-665 significantly reduced luciferase activity of WT HOXB5, but mutation of the miR-665 binding site abrogated the inhibitory effects of miR-665 (right panel). **(B)** The mRNA and protein levels of HOXB5 in EC cells transfected with miR-665 mimic or control mimic were examined using qRT-PCR assay and Western blot analysis. **(C)** The expression of miR-665 in EC cells transfected with miR-665 inhibitor or control inhibitor. **(D)** HEC-1A and HEC-1B cells were transfected as indicated, and the mRNA and protein expression of HOXB5 was investigated using qRT-PCR and Western blot analysis. **(E)** Comparison of HOXB5 expression in EC tissues (*n* = 548) and normal tissues (*n* = 35) (ENCORI database). **(F)** The mRNA expression of HOXB5 in 70 pairs of EC and adjacent normal tissues was checked using qRT-PCR assay. Spearman’s correlation analysis was used to analyze the relationship between HOXB5 expression and DCST1-AS1 or miR-665 expression. **P < 0.01, ***P < 0.001. ^###^*P* < 0.001, compared with si-DCST1-AS1#1 group.

To find out genes sharing the regulatory role of DCST1-AS1 and miR-665, we predicted the target genes of miR-665 using the DIANA database. Among the predicted genes, HOXB5 could be potentially affected by miR-665 (**Figure 4A**). Our luciferase reporter assay demonstrated that overexpression of miR-665 suppressed the luciferase activity of the wild-type HOXB5 3′-UTR luciferase reporter, while it failed to inhibit the luciferase activity of the mutant HOXB5 3′-UTR (**Figure 4A**). Western blot analysis suggested that overexpression of miR-665 inhibited the mRNA and protein expression of HOXB5 (**Figure 4B**), suggesting that HOXB5 is the direct target gene of miR-665.

Since DCTS1-AS1 acts as a sponge of miR-665, we tried to examine whether DCTS1-AS1 could regulate HOXB5 expression *via* miR-665. We transfected EC cells with DCTS1-AS1 siRNA (or control siRNA), along with miR-665 inhibitor (or control inhibitor), and investigated the mRNA and protein levels of HOXB5 using qRT-PCR and Western blot assays. As shown in **Figures 4C, D**, silencing of DCST1-AS1 significantly decreased HOXB5 mRNA and protein expression, whereas inhibition of miR-665 rescued the expression of HOXB5. Our meta-analysis and qRT-PCR assays demonstrated that HOXB5 was dramatically upregulated in EC tissues compared with normal tissues (**Figure 4E**). We also detected a negative correlation between miR-665 and HOXB5 expression and a positive correlation between DCST1-AS1 and HOXB5 expression in EC tissues (**Figure 4F**). These findings suggested that DCST1-AS1 sponges miR-665 to increase HOXB5 expression in EC cells.

### Knockdown of LncRNA DCST1-AS1 Decreases CADM1 Expression *via* Sponging miR-873-5p

**Figure 5:**
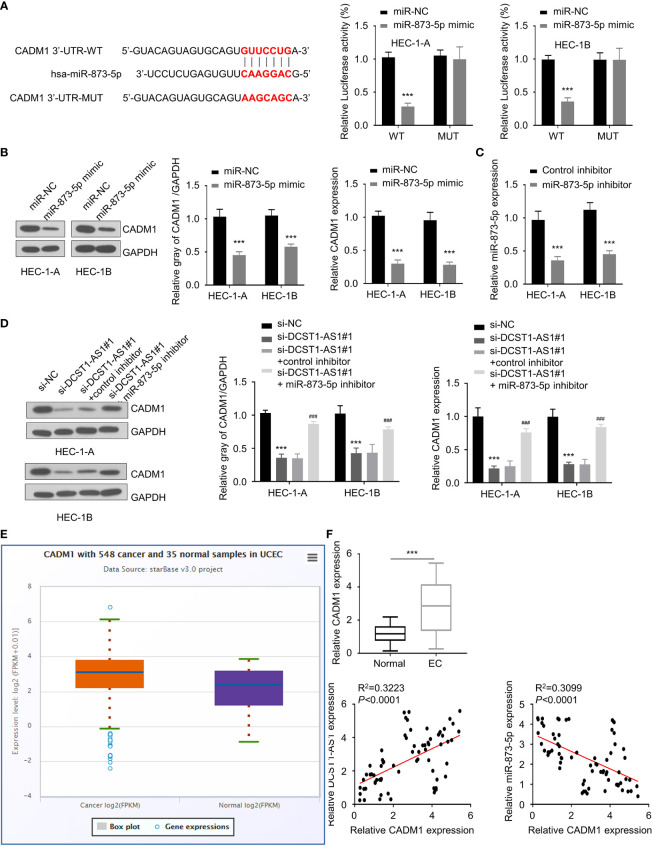
Knockdown of lncRNA DCST1-AS1 decreases cell adhesion molecule 1 (CADM1) expression *via* sponging miR-873-5p. **(A)** Predicted and mutant (lower panel) sequence alignments of miR-873-5p with CADM1 3′-UTR sequence. Luciferase reporter assays showed that miR-873-5p significantly reduced luciferase activity of WT CADM1, but mutation of the miR-873-5p binding site abrogated the inhibitory effects of miR-873-5p (right panel). **(B)** The mRNA and protein levels of CADM1 in EC cells transfected with miR-873-5p mimic or control mimic were examined using qRT-PCR assay and Western blot analysis. **(C)** The expression of miR-873-5p in EC cells transfected with miR-873-5p inhibitor or control inhibitor. **(D)** HEC-1A and HEC-1B cells were transfected as indicated, and the mRNA and protein expression of CADM1 was investigated using qRT-PCR and Western blot analysis. **(E)** Comparison of CADM1 expression in EC tissues (*n* = 548) and normal tissues (*n* = 35) (ENCORI database). **(F)** The mRNA expression of CADM1 in 70 pairs of EC and adjacent normal tissues was checked using qRT-PCR assay. Spearman’s correlation analysis was used to analyze the relationship between CADM1 expression and DCST1-AS1 or miR-873-5p expression. ***P < 0.001. ^###^*P* < 0.001, compared with si-DCST1-AS1#1 group.

Similarly, using an online database, we found that CADM1 is a potential target gene of miR-873-5p (**Figure 5A**). The results from luciferase assays suggested a direct interaction between miR-873-5p and CADM1 3′-UTR (**Figure 5A**). Furthermore, we found that overexpression of miR-873-5p significantly decreased the mRNA and protein levels of CADM1 (**Figure 5B**). Our rescue experiments supported that knockdown of DCST1-AS1 downregulated the expression of CADM1 in EC cells, while inhibition of miR-873-5p had the opposite effects (**Figures 5C, D****)**. Meanwhile, CADM1 was found to be significantly upregulated in EC tissues (**Figures 5E, F****)**. The mRNA level of CADM1 was inversely correlated with miR-873-5p expression, but positively correlated with DCST1-AS1 expression in EC tissues (**Figure 5F**). These results suggested that DCST1-AS1 induces CADM1 expression in EC cells through sponging miR-873-5p.

### LncRNA DCST1-AS1 Promotes EC Progression *via* the miR-665/HOXB5 Signaling

**Figure 6:**
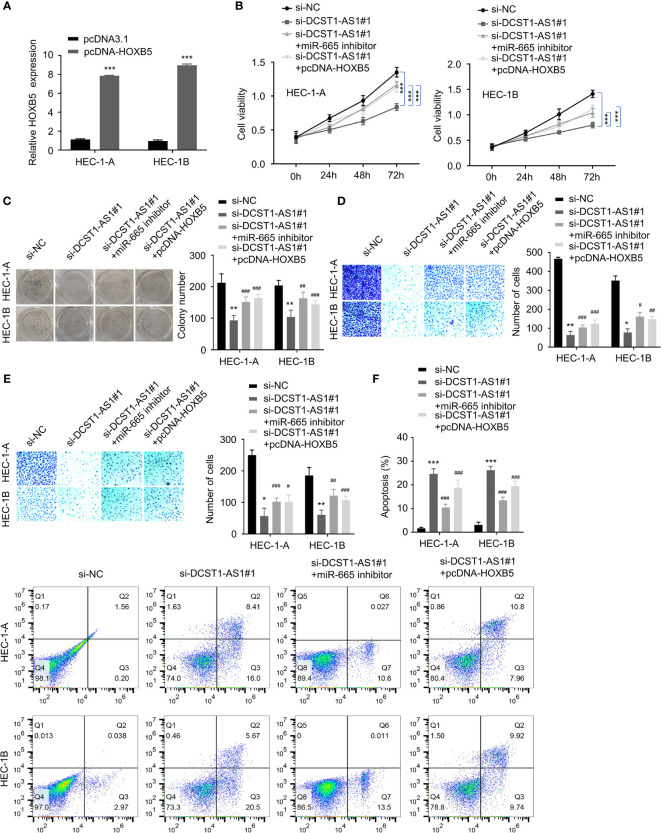
LncRNA DCST1-AS1 promotes EC progression *via* the miR-665/HOXB5 signaling. **(A)** qRT-PCR analysis of HOXB5 expression in EC cells transfected with HXOB5 overexpression plasmid or control plasmid. **(B)** CCK-8 assay showed that either miR-665 inhibition or HOXB5 overexpression rescued EC cell proliferative reduced by DCST1-AS1 knockdown. **(C)** Colony formation assay of EC cells transfected as indicated. **(D, E)** Transwell migration and invasion assay showed that either miR-665 inhibition or HOXB5 overexpression rescued EC cell migration **(D)** and invasion **(E)** that was suppressed by DCST1-AS1 depletion. **(F)** HEC-1A and HEC-1B cells were transfected as indicated, and cell apoptosis was determined by FACS analysis. *P < 0.05, **P < 0.01, ***P < 0.001. ^#^*P* < 0.05, ^##^*P* < 0.01, ^###^*P* < 0.001, compared with si-DCST1-AS1#1 group.

HOXB5, a member of the HOX gene cluster, is a key regulator of developmental processes (24), and it is implicated in multiple human cancers including breast cancer, head and neck cancer, and bladder cancer (25–27). Since we have shown that DCST1-AS1 promotes HOXB5 expression by acting as a sponge for miR-665, we asked whether DCST1-AS1 facilitates EC progression by regulating the miR-665/HOXB5 signaling. To this end, we constructed a HOXB5 overexpression plasmid (**Figure 6A**).

Using CCK-8, colony formation, migration, and invasion assays, we confirmed that silencing of DCST1-AS1 suppressed EC cell proliferation, growth, migration, and invasion, whereas either miR-665 knockdown or overexpression of HOXB5 could reverse the effects of DCST1-AS1 silencing (**Figures 6B–E**). In addition, depletion of DCST1-AS1 increased EC cell apoptosis; however, cell apoptosis was reduced by the transfection with miR-665 inhibitor or HOXB5 overexpression plasmid (**Figure 6F**). These data suggested that DCST1-AS1 promotes EC cell proliferation, migration, and invasion and inhibited cell apoptosis, at least in part, by regulating the miR-665/HOXB5 pathway.

### LncRNA DCST1-AS1 Promotes the Malignant Features of EC Cells Through the miR-873-5p/CADM1 Signaling

**Figure 7:**
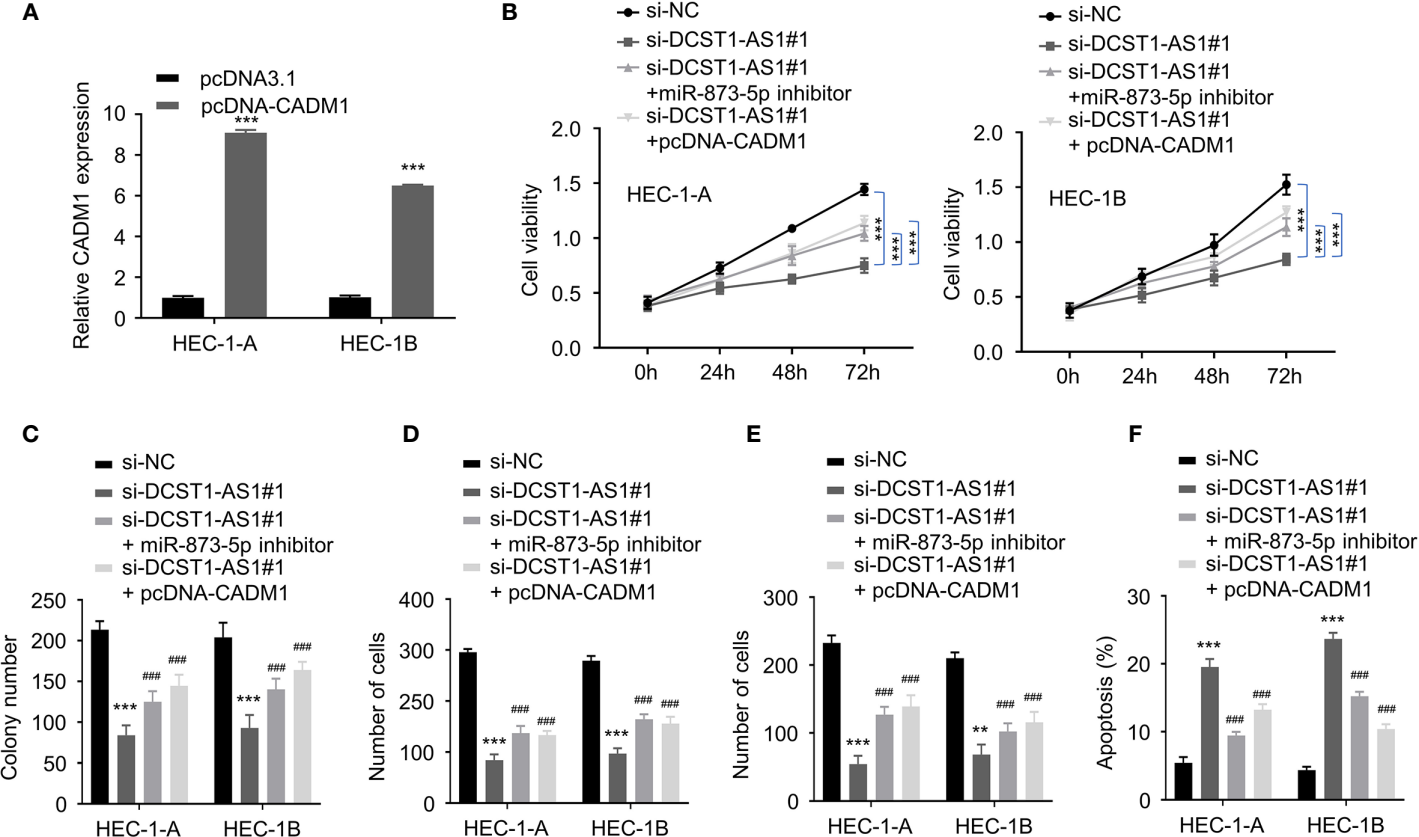
LncRNA DCST1-AS1 promotes the malignant features of EC cells through miR-873-5p/CADM1 signaling. **(A)** qRT-PCR analysis of CADM1 expression in EC cells transfected with CADM1 overexpression plasmid or control plasmid. **(B)** CCK-8 assay showed that either miR-873-5p inhibition or CADM1 overexpression rescued EC cell proliferative reduced by DCST1-AS1 knockdown. **(C)** Colony formation assay of EC cells transfected as indicated. **(D, E)** Transwell migration and invasion assay showed that either miR-6873-5p inhibition or CADM1 overexpression rescued EC cell migration **(D)** and invasion **(E)** that was suppressed by DCST1-AS1 depletion. **(F)** HEC-1A and HEC-1B cells were transfected as indicated, and cell apoptosis was determined by FACS analysis. **P < 0.01, ***P < 0.001. ^###^*P* < 0.001, compared with siDCST1-AS1#1 group.

To explore whether the miR-873-5p/CADM1 axis mediates the function of DCST1-AS1 in EC cells, we constructed a CADM1 overexpression plasmid (**Figure 7A**). Our rescue experiments revealed that suppression of DCST1-AS1 significantly suppressed EC cell proliferation, migration, and invasion and induced cell apoptosis (**Figures 7B–F**). In contrast, either knockdown of miR-873-5p or CADM1 overexpression rescued the malignant features of EC cells and reduced cell apoptosis (**Figures 7B–F**). These data indicated that DCST1-AS1 promotes the aggressive phenotype in EC cells, at least in part, by regulating the miR-873-5p/CADM1 pathway.

### LncRNA DCST1-AS1 Promotes EC Tumor Growth *In Vivo*

**Figure 8:**
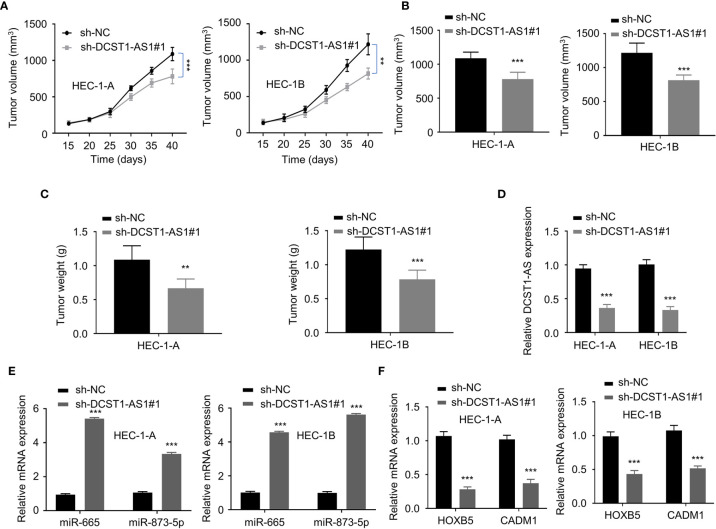
LncRNA DCST1-AS1 promotes EC tumor growth *in vivo*. **(A, B)** The growth curves **(A)** and tumor volume **(B)** after injection of DCST1-AS1 shRNA#1 or control shRNA. **(C)** The weight of tumors. **(D)** The expression of DCST1-AS1 was detected in tumors from control shRNA or DCST1-AS1 shRNA#1 group mice by qRT-PCR assay. **(E)** The expression of miR-665 and miR-873-5p in tumor samples was detected by RT-qPCR assay. **(F)** The qRT-PCR assay of HOXB5 and CADM1 expression in tumor samples from different groups. **P < 0.01, ***P < 0.001.

To validate the effects of lncRNA DCST1-AS1 on tumor growth *in vivo*, we injected EC cells transfected with DCST1-AS1 shRNA or control shRNA into nude mice. The results from the tumor xenografts in nude mice showed that the volume and weight of subcutaneous tumors were significantly suppressed by DCST1-AS1 knockdown (**Figures 8A–C**). Our qRT-PCR analysis of the tumors suggested that knockdown of DCST1-AS1 significantly increased the expression of miR-665 and miR-873-5p, but decreased the expression of HOXB5 and CADM1 (**Figures 8D–F**). Taken together, our results suggested that DCST1-AS1 promotes EC growth *in vivo*.

## Discussion

LncRNAs are considered as critical epigenetic regulators, and the aberrant expression of lncRNAs contributes to cancer progression (28, 29). The effect of lncRNA DCST1-AS1 has been investigated in multiple human tumors, such as breast cancer, cervical cancer, hepatocellular carcinoma, and glioblastoma (17–19, 30, 31). However, the function of DCST1-AS1 in EC is largely unknown. In the present study, we demonstrated that DCST1-AS1 serves as an oncogenic lncRNA in EC (20). Mechanically, we reported that DCST1-AS1 functions as a sponge for miR-665 and miR-873-5p and subsequently upregulates the expression of HOXB5 and CADM1, respectively.

Previous studies have shown that miR-665 could play oncogenic or tumor-suppressive roles in various types of cancer. For instance, miR-665 was shown to act as an oncogene in ovarian cancer by directly inhibiting SRC kinase signaling inhibitor 1 (SRCIN1) expression (32). Elevated expression of miR-665 was associated with poor prognosis in nonsmall cell lung cancer (33). In breast cancer, miR-665 represses the expression of nuclear receptor subfamily 4 group A member 3 (NR4A3) to activate the MAPK/ERK kinase signaling (34). In contrast, the tumor-suppressor role of miR-665 has been also reported. In osteosarcoma, miR-665 regulates Rab23 expression to inhibit tumor invasion and metastasis (35). Additionally, miR-665 suppresses epithelial–mesenchymal transition (EMT) and gastric cancer progression by targeting cysteine-rich motor neuron protein 1 (CRIM1) (36). However, little is known about the function of miR-665 in EC as well as the association between lncRNA DCST1-AS1 with miR-665. Here, we demonstrated that miR-665 could inhibit the proliferation and invasiveness of EC cells, and the DST1-AS1/miR-665 axis regulates EC development. Moreover, we screened the target genes of miR-665 and confirmed that miR-665 directly targets HOXB5, which functions as a transcription factor in several cancer types (37). The tumor-promoting roles of HOXB5 have been found in breast cancer, gastric carcinoma, lung cancer, retinoblastoma, and neck squamous cell carcinoma (26, 27, 38–41). In this study, we demonstrated for the first time that HOXB5 could enhance EC cell proliferation and invasiveness. We also revealed that DCST1-AS1 exerts oncogenic functions partly *via* sponging miR-665 and by upregulating HOXB5 expression in EC.

Using the online predicting database, we identified that miR-873-5p is another potential target of DCST1-AS1. Accumulating studies have shown that miR-873-5p represses tumor progression in various human cancers. For example, miR-873-5p was downregulated in colorectal cancer and overexpression of miR-873-5p represses cancer cell migration, invasion, and EMT through targeting ZEB1 (42, 43). Moreover, miR-873-5p could regulate chemokine (C-X-C motif) ligand 16 (CXCL16) expression to inhibit thyroid cancer progression (44). Besides, miR-873-5p was reported to reduce gastric cancer cell proliferation by mediating hedgehog-GLI signaling (22). In glioblastoma, miR-873-5p repressed IGF2 mRNA-binding protein 1 (IGF2BP1) expression to suppress glioblastoma tumorigenesis (45). MiR-873-5p showed tumor-suppressive effects in esophageal cancer *via* modulating the miR-873/DEC2 axis (46). In contrast, there is also evidence showing that miR-873-5p functions as an oncogene in hepatocellular carcinoma and lung adenocarcinoma (47, 48). In endometrial cancer, miR-873-5p exerts a tumor-suppressor role *via* directly targeting hepatoma−derived growth factor (HDGF) (49). Consistent with this report, we demonstrated that miR-873-5p expression was reduced in EC tissues and further proved that miR-873-5p functions as a key tumor suppressor and a downstream target of DCST1-AS1 in EC cells. CADM1 is a member of single transmembrane glycoproteins that belong to the immunoglobulin superfamily involved in synapse formation and plasticity (50, 51). CADM1 was frequently reported as a tumor suppressor and mostly was abrogated in various cancer types. Loss of CADM1 expression predicted poor prognosis and the development of esophageal cancer and ovarian cancer (52, 53). Also, CADM1 exerts its tumor-suppressor effects in breast cancer, bladder cancer, and ovarian cancer (54–56). However, we revealed that CADM1 is overexpressed in EC and it promotes EC progression.

This study not only advances our understanding of the roles of the DCST1-AS1/miR-665/HOXB5 pathway and DCST1-AS1/miR-873-5p/CADM1 pathway in EC biology but also provides these signaling pathways as new targets for developing therapy of EC in the future.

## Data Availability Statement

The original contributions presented in the study are included in the article/supplementary material. Further inquiries can be directed to the corresponding author.

## Ethics Statement

The studies involving human participants were reviewed and approved by the Ethical Committee on Scientific Research of People’s Hospital of Rizhao. The patients/participants provided their written informed consent to participate in this study.

## Author Contributions

PS and JW designed and conducted the experiments. HT, LL, HG, and XW analyzed the data. JW wrote the manuscript and PS revised the manuscript. All authors contributed to the article and approved the submitted version.

## Conflict of Interest

The authors declare that the research was conducted in the absence of any commercial or financial relationships that could be construed as a potential conflict of interest.

## Publisher’s Note

All claims expressed in this article are solely those of the authors and do not necessarily represent those of their affiliated organizations, or those of the publisher, the editors and the reviewers. Any product that may be evaluated in this article, or claim that may be made by its manufacturer, is not guaranteed or endorsed by the publisher.

## Abbreviations

EC, endometrial cancer; lncRNAs, long noncoding RNAs; DCST1-AS1, DC-STAMP domain-containing 1-antisense 1; HESCs, human normal endometrial stromal cells; NC, negative control.
