# Corrigendum: LncRNA DCST1-AS1 Promotes Endometrial Cancer Progression by Modulating the MiR- 665/HOXB5 and MiR-873-5p/CADM1 Pathways

**DOI:** 10.3389/fonc.2022.819908

**Published:** 2022-03-29

**Authors:** Jie Wang, Pingping Shi, Huaixiang Teng, Lixiang Lu, Hailong Guo, Xiuqin Wang

**Affiliations:** ^1^Gynaecology Clinic, People’s Hospital of Rizhao, Rizhao, China; ^2^No. 2 Department of Gynaecology, People’s Hospital of Rizhao, Rizhao, China; ^3^Reproductive Medicine Center, Maternal and Child Health Hospital of Rizhao, Rizhao, China; ^4^No. 2 Department of Gynaecology, Baiqiuen Hospital of Rizhao, Rizhao, China

**Keywords:** endometrial cancer, long noncoding RNA, DC-STAMP domain-containing 1-antisense 1, microRNA-665, homeobox B5, microRNA-873-5p, cell adhesion molecule 1

In the original article, Changjiang Lei was designated as one of the authors. Changjiang Lei has now been removed from the author list. The corrected Author Contributions Statement appears below.

The authors apologize for this error and state that this does not change the scientific conclusions of the article in any way. The original article has been updated.

## Author Contributions

PS and JW designed and conducted the experiments. HT, LL, HG, and XW analyzed the data. JW wrote the manuscript and PS revised the manuscript. All authors contributed to the article and approved the submitted version.

## Publisher’s Note

All claims expressed in this article are solely those of the authors and do not necessarily represent those of their affiliated organizations, or those of the publisher, the editors and the reviewers. Any product that may be evaluated in this article, or claim that may be made by its manufacturer, is not guaranteed or endorsed by the publisher.

